# Supplementary material for: Profiling of Extracellular Small RNAs Highlights a Strong Bias towards Non-Vesicular Secretion
Source: Cells. 2021 Jun 18;10(6):1543. doi: 10.3390/cells10061543 (PMC8235078; doi:10.3390/cells10061543)
Supplement: Supplementary file 1 [file cells-10-01543-s001.zip › cells-1238532-supplementary.pdf]

SUPPLEMENTARY FIGURES

Profiling of Extracellular Small RNAs Highlights a Strong Bias towards Non-Vesicular Secretion

Helena Sork, Mariana Conceicao, Giulia Corso, Joel Nordin, Yi Xin Fiona Lee, Kaarel Krjutskov, Jakub Orzechowski Westholm, Pieter Vader, Marie Pauwels, Roosmarijn E. Vandenbroucke, Matthew JA Wood, Samir EL Andaloussi and Imre Mäger

---

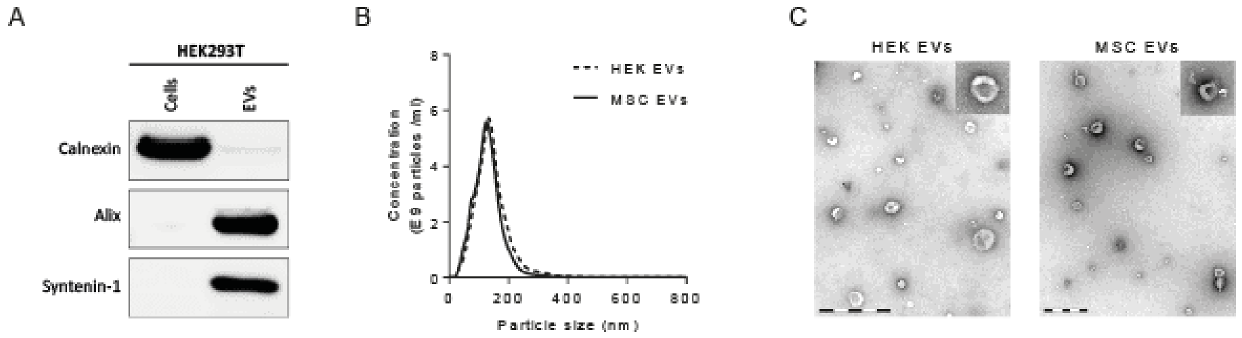

Supplementary Figure S1. EV characterization (A) A representative Western blot demonstrating the presence of EV marker proteins Alix and Syntenin-1 in isolated EV samples. (B) Representative nanoparticle tracking analysis profiles demonstrating the size distribution profile of isolated EV samples. (C) Representative electron microscopy images demonstrating the typical EV size and morphology in isolated samples. Scale bar 500nm.

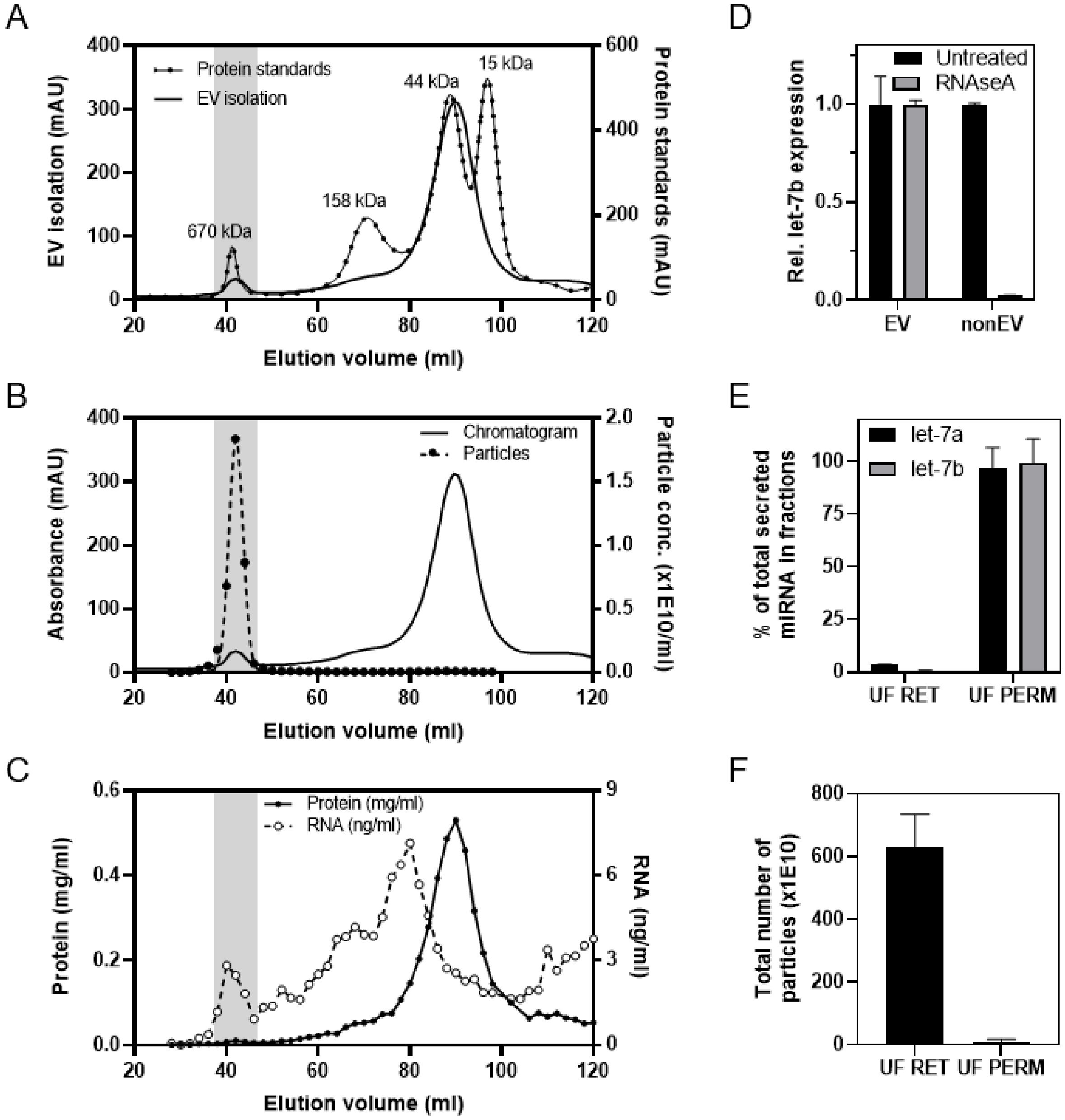

Supplementary Figure S2. Further validation of the SEC workflow for separating EV and non-EV secreted material using Neuro2a cells after overexpression of let-7a miRNA. The SEC chromatograms (A, B, C) indicate that the EV peak (i.e. the void volume peak) contains material > 600 kDa MW and is the only fraction to contain particles, as detected by the Nanoparticle Tracking Analysis. The elution profile of RNA and protein (C) is more complex, where a significant proportion of material is found in the non-EV area of the chromatogram. (D) miRNA in the EV fraction is protected from RNAseA treatment, whereas the non-EV fraction was susceptible for that. (E) When the conditioned medium is concentrated with 100 kDa MWCO spin filters the majority of miRNA is found in the ultrafiltration permeate (UF PERM), which is consistent with the observation that the majority of miRNAs are found in the non-EV peak of the SEC workflow. Ultrafiltration retentate (UF RET) contained only a minor fraction of miRNA). (F) No particles were detected in the UF permeate, again in line with the observation that the majority of miRNA is found in the non-EV fraction.

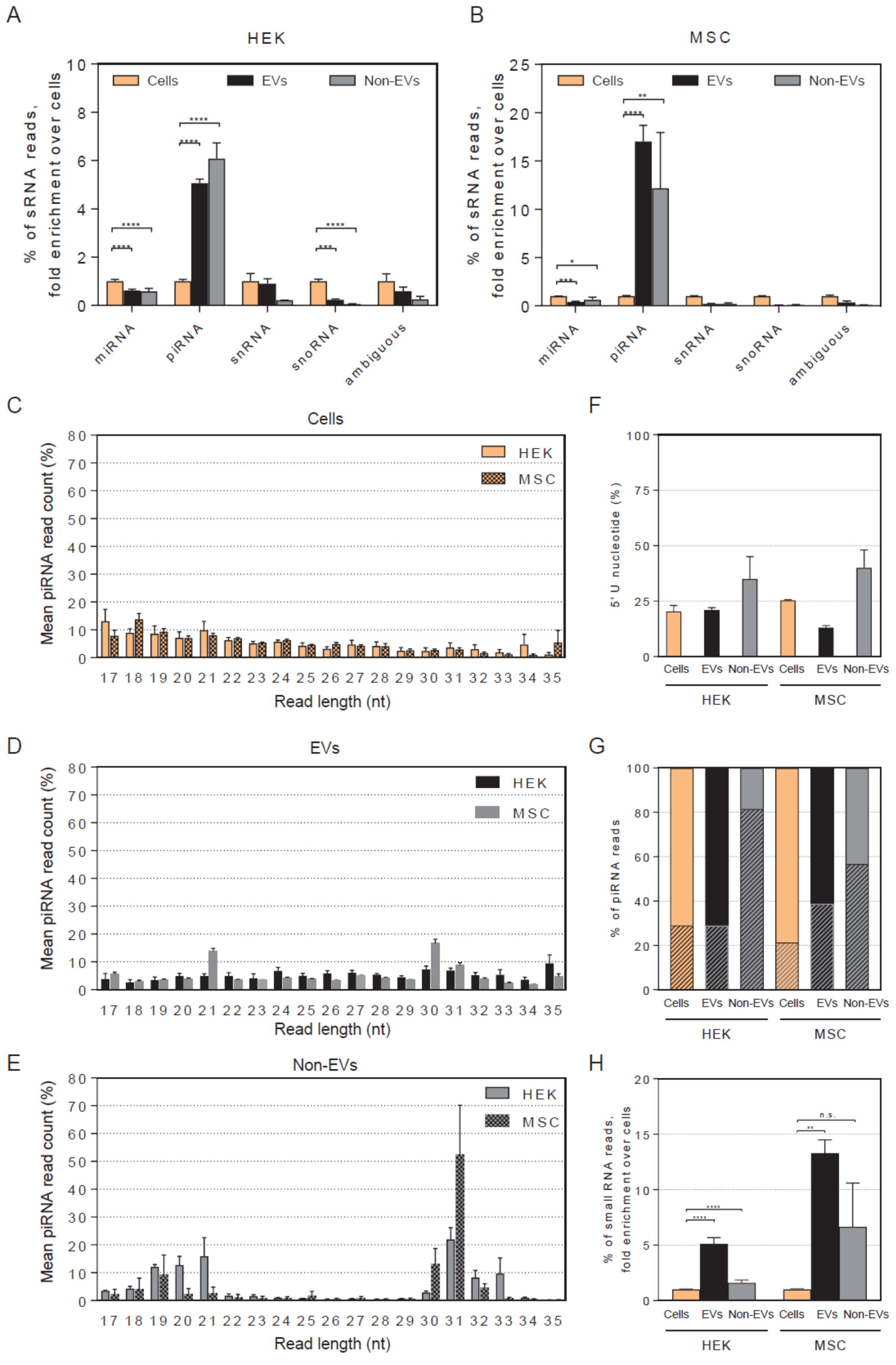
A
HEK
% of sRNA reads, fold enrichment over cells
Cells EVs Non-EVs
miRNA piRNA snRNA snoRNA ambiguous
B
MSC
% of sRNA reads, fold enrichment over cells
Cells EVs Non-EVs
miRNA piRNA snRNA snoRNA ambiguous
C
Cells
Mean piRNA read count (%)
HEK MSC
Read length (nt)
D
EVs
Mean piRNA read count (%)
HEK MSC
Read length (nt)
E
Non-EVs
Mean piRNA read count (%)
HEK MSC
Read length (nt)
F
5' U nucleotide (%)
Cells EVs Non-EVs Cells EVs Non-EVs
HEK MSC
G
% of piRNA reads
Cells EVs Non-EVs Cells EVs Non-EVs
HEK MSC
H
% of small RNA reads, fold enrichment over cells
n.s.
Cells EVs Non-EVs Cells EVs Non-EVs
HEK MSC

Supplementary Figure S3. sRNA composition, characteristics and enrichment in secretory fractions. The percentage of sRNA biotype reads, fold enrichment over cells in HEK (A) and MSC (B) samples reveals that EV and Non-EV samples are enriched in piRNA sequences and depleted from miRNA sequences. piRNA reads distribution length in cells (C), EVs (D) and Non-EVs (E), and the presence of 5’ U (F) suggests that a proportion of genes annotated as piRNAs might not be true piRNAs. Up to a third of piRNA annotations in cells and EVs, and up to 80% of piRNA annotations in Non-EVs also align to tRNA reads (G). Even if the tRNA-overlapping piRNA sequences (dashed regions on the bars) are eliminated from the analysis, the enrichment of piRNA sequences in EVs and Non-EVs is still observed (H). Two-way ANOVA analysis with Tukey post-hoc correction. **$p<0.01$, *** $p<0.001$, **** $p<0.0001$

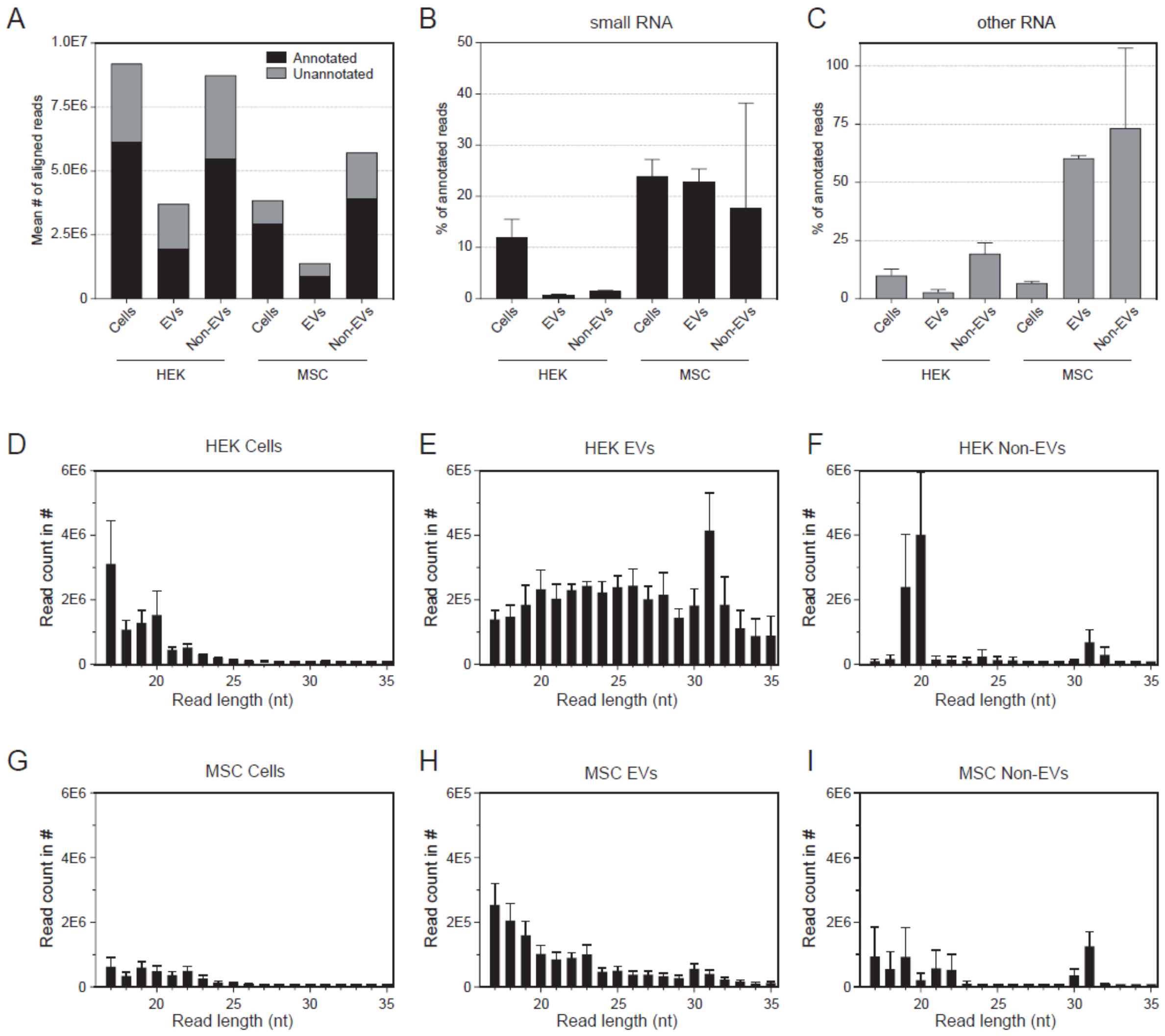

Supplementary Figure S4. Read alignment, annotation and length statistics across the studies samples. The average number of aligned reads varies between sample types (A). The percentage of annotated reads that align to sRNA biotype RNAs and other RNA sequences is presented in panels (B) and (C), respectively (respective rRNA data is not presented). Length distribution histograms of all annotated reads (sRNA, rRNA and other RNA) for HEK and MSC cells, EVs and Non-EVs is presented in panels (D–I).

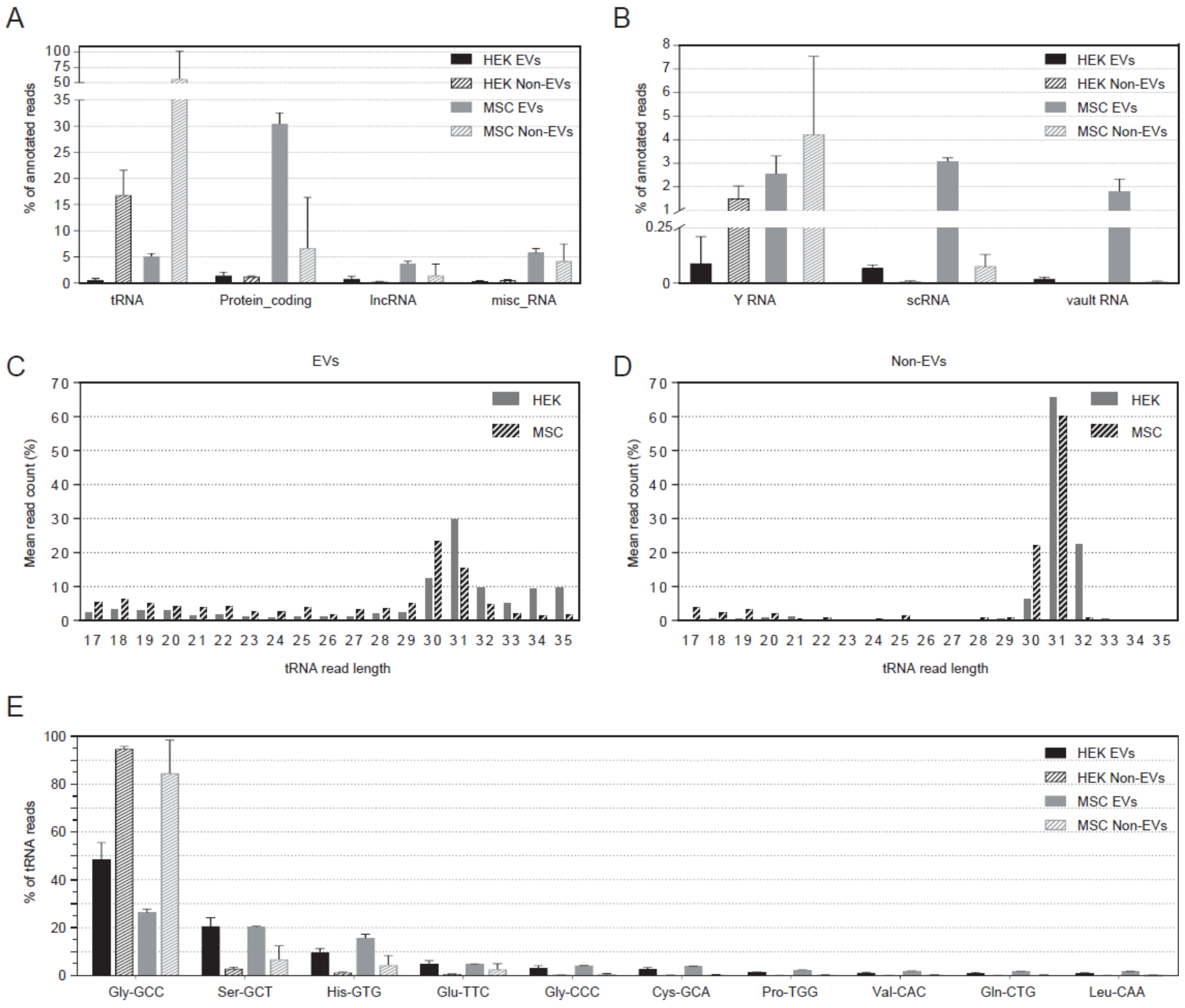

Supplementary Figure S5. The prevalence of 'other RNA' biotypes (A) The percentage of annotated reads aligned to tRNA, protein coding RNA, long non-coding RNA (lncRNA) and miscellaneous RNA (misc_RNA). (B) The percentage of annotated reads aligned to Y RNA, small cytoplasmic RNA (scRNA) and vault RNA. (C, D) Read length distribution histograms of tRNA-aligned reads in HEK and MSC EVs and Non-EVs. (E) Among the tRNA-aligning reads, Gly-GCC tRNA sequences are overrepresented, constituting up to 90% of all detected RNA reads in the Non-EV samples.

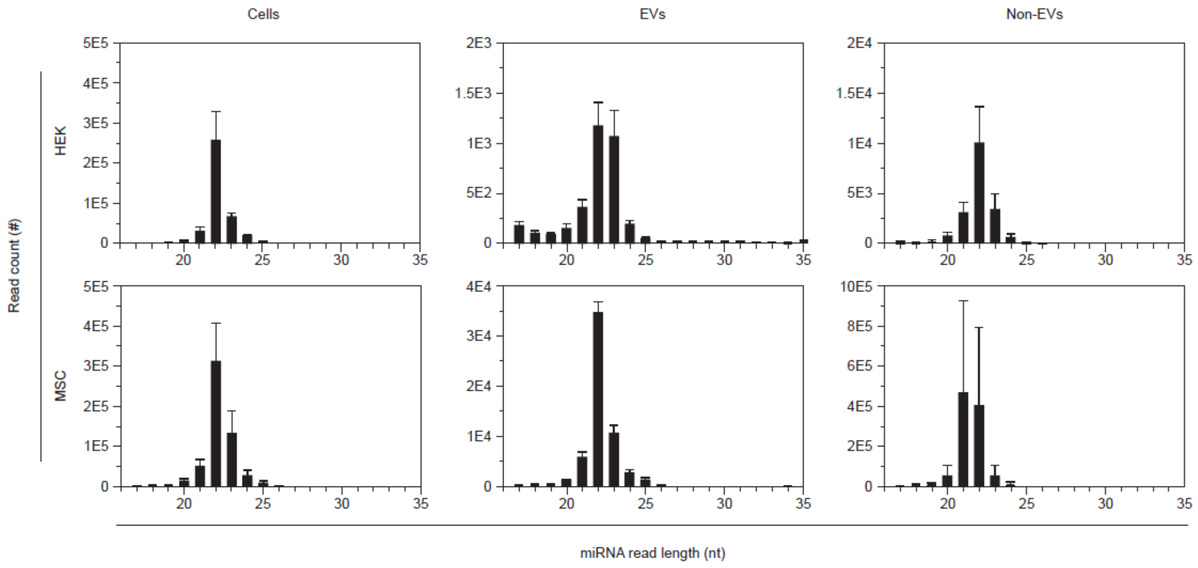

Supplementary Figure S6. Length distribution histograms for reads aligning to miRNAs. Sequences in all samples are in accordance with an expected length of mature miRNAs.

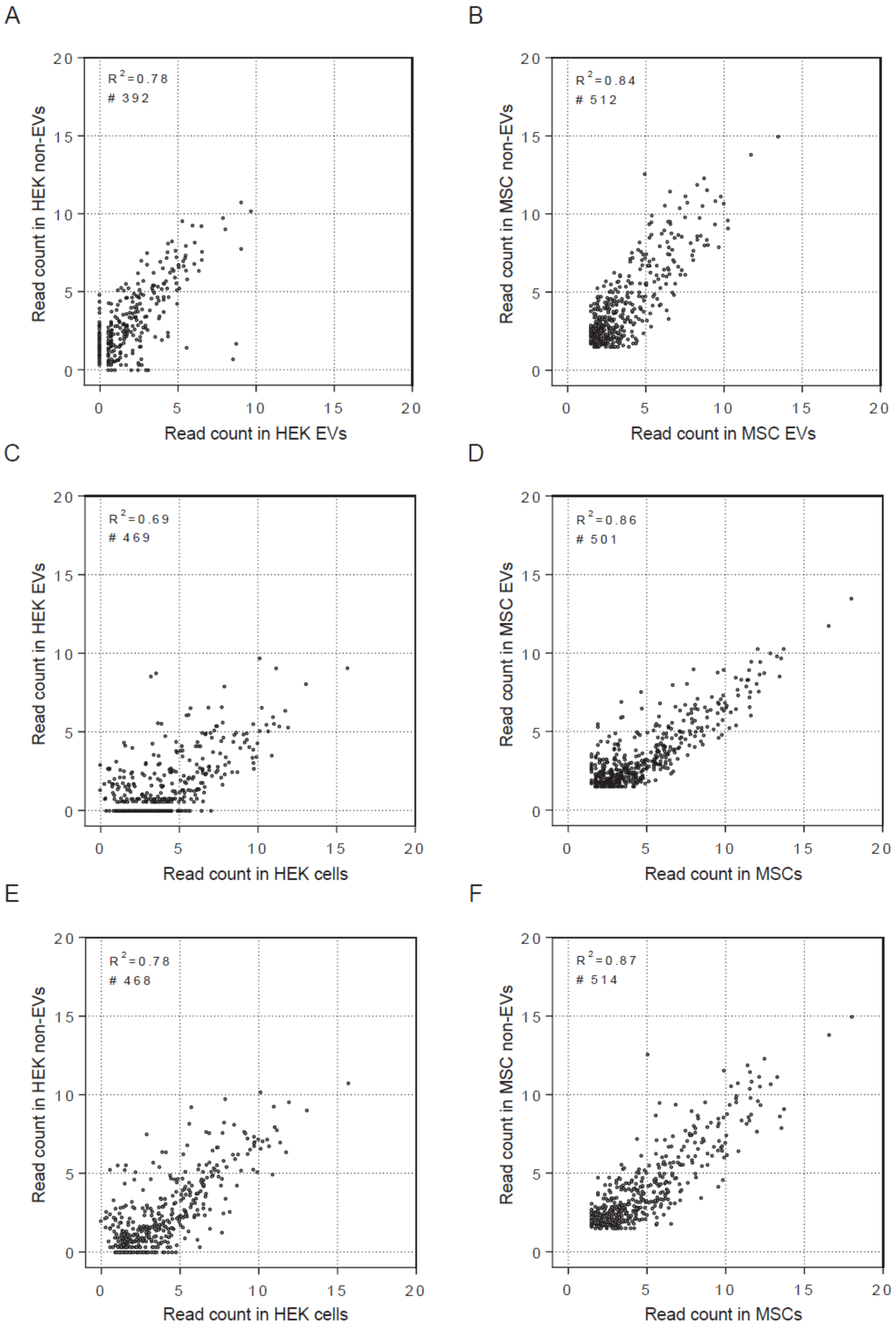

Supplementary Figure S7. Correlation plots of all mature miRNAs. miRNA expression profile between EV, Non-EV and cell samples are highly correlated both in HEK (A, C, E) and MSC (B, D, F) samples.

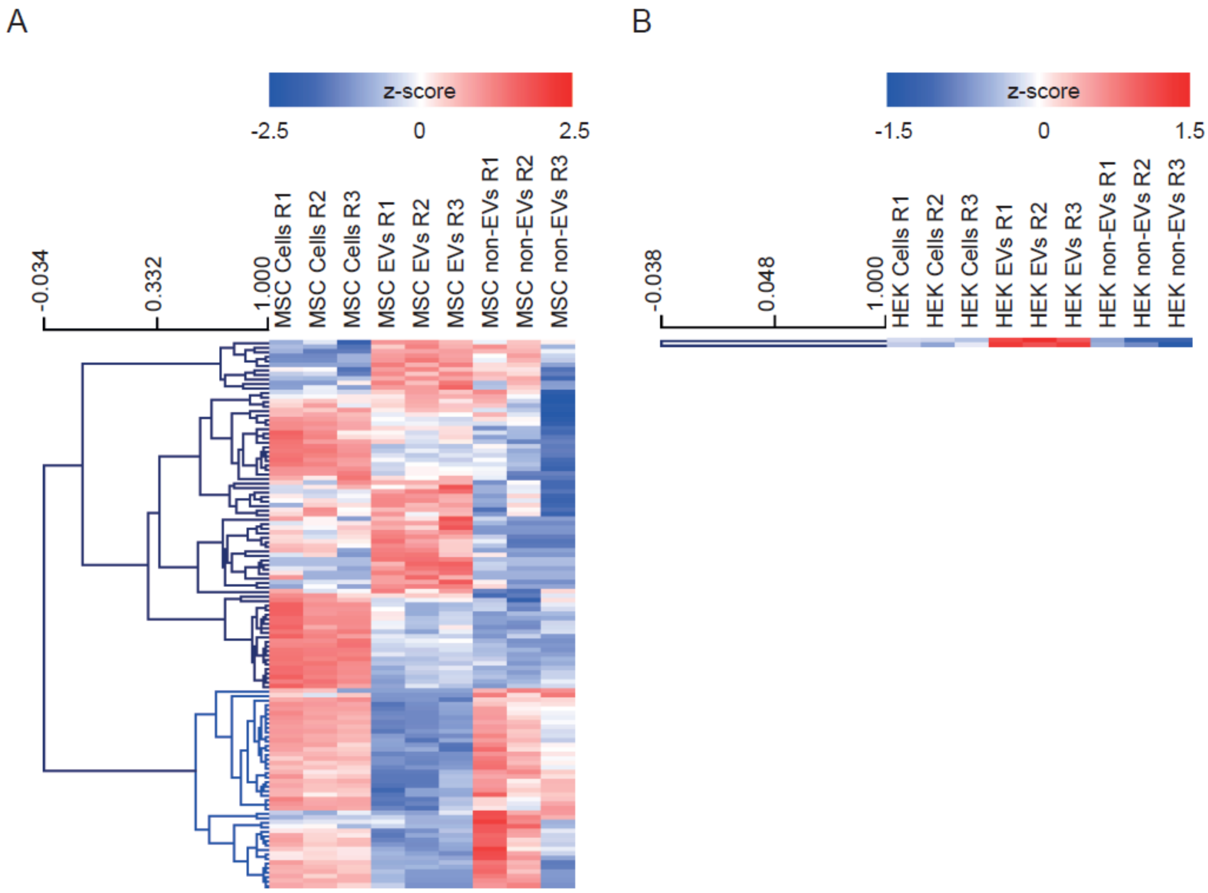

Supplementary Figure S8. Heatmaps of differentially expressed miRNAs detected between the EV and Non-EV fraction of the MSC (A) and HEK (B) secretome. EV-enriched miRNAs tend to have low expression level in cells, whereas EV-depleted miRNAs are sampled from miRNAs with high cellular expression. In HEK samples, there are only two miRNAs that are differentially expressed between EV and Non-EV samples (B), whereas in MSC samples the number of differentially expressed miRNAs is considerably higher.

Supplementary Table S1. Differentially expressed miRNAs in EVs *vs* Cells.

EV *versus* cells									
	HEK					MSC			
DE miRNA name	log2FC	lfcSE	pvalue	padj	DE miRNA name	log2FC	lfcSE	pvalue	padj
hsa-miR-7704	7.9	1.0	3.34E-14	9.97E-12	hsa-miR-6892-5p	7.1	0.7	4.5E-16	3.4E-14
hsa-miR-6087	7.3	1.0	1.21E-12	1.80E-10	hsa-miR-365a-5p	6.4	0.7	2.0E-18	3.8E-16
hsa-miR-4492	6.0	1.2	1.74E-06	6.47E-05	hsa-miR-128-1-5p	6.2	0.9	4.1E-10	1.5E-08
hsa-miR-4497	5.9	1.2	4.87E-07	3.63E-05	hsa-miR-503-3p	6.2	1.0	1.4E-07	2.6E-06
hsa-miR-3610	5.8	1.1	1.26E-06	6.28E-05	hsa-miR-193b-5p	5.7	0.7	1.4E-17	1.4E-15
hsa-miR-4508	5.5	1.2	1.64E-06	6.47E-05	hsa-miR-451a	5.7	0.9	3.1E-09	9.1E-08
hsa-miR-4792	5.4	1.1	1.40E-08	1.39E-06	hsa-miR-365b-5p	5.7	0.6	9.9E-20	3.8E-17
hsa-miR-1225-3p	4.9	1.3	1.45E-04	3.09E-03	hsa-miR-6807-5p	5.6	1.0	2.5E-07	4.4E-06
hsa-miR-149-3p	4.5	1.3	3.85E-04	6.75E-03	hsa-miR-1307-5p	5.5	0.6	3.9E-18	5.1E-16
hsa-miR-199a-3p	4.3	0.9	1.15E-06	6.28E-05	hsa-miR-323a-5p	5.4	0.9	2.4E-08	5.5E-07
hsa-miR-3648	4.2	1.1	2.27E-06	7.52E-05	hsa-miR-152-5p	5.3	0.7	1.2E-11	5.3E-10
hsa-miR-654-5p	4.0	1.3	8.13E-05	2.02E-03	hsa-miR-1225-3p	5.1	1.0	1.2E-05	1.4E-04
hsa-miR-199b-3p	3.7	0.9	3.49E-05	1.04E-03	hsa-miR-410-5p	4.9	1.0	1.5E-05	1.8E-04
hsa-miR-4787-3p	3.5	1.2	4.04E-03	3.65E-02	hsa-miR-23a-5p	4.9	0.8	8.6E-10	2.8E-08
hsa-miR-4516	3.5	1.3	3.52E-04	6.55E-03	hsa-miR-433-5p	4.8	1.1	1.1E-05	1.4E-04
hsa-miR-365b-5p	3.5	1.2	3.66E-03	3.64E-02	hsa-miR-7977	4.6	0.8	2.4E-08	5.5E-07
hsa-miR-940	3.5	1.2	2.00E-03	2.29E-02	hsa-miR-6892-3p	4.6	1.2	2.7E-04	2.2E-03
hsa-miR-143-5p	3.4	1.3	4.19E-03	3.67E-02	hsa-miR-142-5p	4.5	0.9	1.3E-05	1.5E-04
hsa-miR-3195	3.4	1.5	5.61E-03	4.40E-02	hsa-miR-92a-1-5p	4.5	0.8	2.9E-08	6.3E-07
hsa-miR-146a-5p	3.3	1.0	7.47E-04	1.11E-02	hsa-miR-4669	4.5	1.0	1.6E-05	1.8E-04
hsa-let-7b-5p	3.3	0.9	8.08E-05	2.02E-03	hsa-miR-370-5p	4.4	0.8	1.2E-07	2.3E-06
hsa-miR-214-3p	3.2	1.2	2.94E-03	3.22E-02	hsa-miR-6789-3p	4.4	1.1	5.8E-04	4.0E-03
hsa-miR-4532	3.2	1.2	1.45E-04	3.09E-03	hsa-miR-1275	4.3	0.8	6.0E-07	9.6E-06
hsa-miR-379-5p	3.2	1.1	1.91E-03	2.27E-02	hsa-miR-149-3p	4.2	1.0	1.8E-04	1.5E-03
hsa-miR-125b-1-3p	3.1	1.1	3.02E-03	3.22E-02	hsa-miR-1226-5p	4.2	1.2	4.1E-04	3.0E-03
hsa-miR-143-3p	3.0	1.0	6.51E-04	1.02E-02	hsa-miR-194-3p	4.1	1.2	1.2E-03	7.9E-03
hsa-miR-206	2.9	1.0	1.51E-03	1.87E-02	hsa-miR-431-3p	4.0	0.8	5.3E-07	8.8E-06
hsa-miR-21-5p	2.5	0.9	3.99E-03	3.65E-02	hsa-miR-3187-3p	3.9	0.9	4.4E-05	4.1E-04
hsa-miR-100-5p	2.5	0.9	3.96E-03	3.65E-02	hsa-miR-329-5p	3.8	1.1	2.1E-03	1.2E-02
hsa-miR-199a-5p	2.5	0.9	5.00E-03	4.03E-02	hsa-miR-487b-5p	3.8	1.0	7.9E-04	5.3E-03
hsa-let-7i-5p	2.4	0.9	4.96E-03	4.03E-02	hsa-miR-5100	3.8	1.0	4.1E-04	3.0E-03
hsa-miR-432-5p	2.3	1.4	3.49E-03	3.58E-02	hsa-miR-92b-5p	3.8	0.6	9.1E-11	3.5E-09
hsa-miR-128-3p	-2.9	0.9	1.40E-03	1.81E-02	hsa-miR-193a-5p	3.7	0.5	6.3E-12	3.5E-10
hsa-miR-103a-3p	-2.9	0.9	8.69E-04	1.23E-02	hsa-miR-23b-5p	3.7	0.8	3.6E-05	3.5E-04
hsa-miR-148a-3p	-3.5	1.0	4.25E-04	7.04E-03	hsa-miR-4443	3.7	0.9	4.5E-05	4.2E-04
hsa-miR-615-3p	-3.6	1.0	3.16E-04	6.28E-03	hsa-miR-486-5p	3.6	0.8	2.4E-06	3.5E-05
hsa-miR-629-5p	-3.7	1.3	4.40E-03	3.75E-02	hsa-miR-4523	3.5	1.2	3.0E-03	1.6E-02
hsa-miR-192-5p	-4.1	1.2	9.12E-04	1.24E-02	hsa-miR-1343-3p	3.4	0.9	2.0E-04	1.6E-03
					hsa-miR-148b-5p	3.4	0.9	4.4E-04	3.1E-03
					hsa-miR-30b-3p	3.3	1.1	5.2E-03	2.7E-02
					hsa-miR-150-5p	3.3	1.2	2.3E-03	1.3E-02
					hsa-miR-4426	3.3	1.3	9.2E-03	4.4E-02
					hsa-miR-654-5p	3.2	0.5	1.2E-11	5.3E-10
					hsa-miR-25-5p	3.2	0.7	7.6E-06	1.0E-04
					hsa-miR-125a-3p	3.2	0.6	9.0E-08	1.8E-06
					hsa-miR-877-5p	3.1	1.3	8.3E-03	4.0E-02
					hsa-miR-3187-5p	3.1	1.3	1.0E-02	4.8E-02
					hsa-miR-29b-1-5p	3.0	1.0	3.7E-03	1.9E-02
					hsa-miR-485-5p	3.0	0.6	1.6E-06	2.5E-05
					hsa-miR-1468-5p	2.9	0.9	1.8E-03	1.1E-02
					hsa-miR-1908-5p	2.8	0.7	3.4E-05	3.3E-04
					hsa-miR-541-3p	2.8	1.0	7.4E-03	3.7E-02
					hsa-miR-1228-5p	2.6	1.1	9.3E-03	4.4E-02
					hsa-miR-377-5p	2.5	0.8	1.6E-03	9.9E-03
					hsa-miR-671-5p	2.4	0.6	2.5E-05	2.6E-04
					hsa-miR-183-5p	2.1	0.8	7.2E-03	3.6E-02
					hsa-miR-1307-3p	2.0	0.5	1.1E-04	9.9E-04
					hsa-miR-148a-3p	2.0	0.6	3.9E-04	3.0E-03
					hsa-miR-574-3p	2.0	0.6	3.1E-04	2.4E-03
					hsa-miR-122-5p	2.0	0.8	8.9E-04	6.0E-03
					hsa-miR-941	1.7	0.6	4.0E-03	2.1E-02
					hsa-miR-99b-3p	1.7	0.6	3.0E-03	1.7E-02
					hsa-miR-25-3p	1.6	0.4	1.6E-04	1.4E-03
					hsa-miR-185-5p	1.5	0.5	7.1E-04	4.9E-03
					hsa-miR-221-5p	1.5	0.4	1.2E-03	8.0E-03
					hsa-miR-99a-5p	-1.5	0.4	6.8E-05	6.1E-04
					hsa-miR-100-5p	-1.6	0.6	8.4E-03	4.1E-02
					hsa-miR-22-3p	-1.7	0.4	6.2E-06	8.5E-05
					hsa-miR-30a-5p	-1.8	0.6	3.3E-03	1.7E-02
					hsa-miR-21-5p	-1.9	0.3	1.9E-08	4.9E-07
					hsa-miR-654-3p	-2.2	0.5	2.2E-05	2.3E-04
					hsa-miR-181a-5p	-2.3	0.5	2.6E-06	3.7E-05
					hsa-miR-29a-3p	-2.3	0.4	1.8E-08	4.9E-07
					hsa-miR-381-3p	-2.5	0.6	1.6E-05	1.8E-04
					hsa-miR-21-3p	-2.6	0.7	3.8E-04	3.0E-03
					hsa-miR-199a-5p	-2.8	0.4	1.5E-12	9.4E-11
					hsa-miR-193b-3p	-3.2	1.0	2.5E-03	1.4E-02
					hsa-miR-136-3p	-3.5	0.8	2.5E-05	2.6E-04

Supplementary Table S2. Differentially expressed miRNAs in Non-EVs *vs* Cells.

non-EV *versus* cells									
DE miRNA name	HEK				DE miRNA name	MSC			
	log2FC	lfcSE	pvalue	padj		log2FC	lfcSE	pvalue	padj
hsa-miR-4532	8.7	1.3	8.01E-10	2.88E-07	hsa-miR-122-5p	8.8	0.8	1.64E-28	5.69E-26
hsa-miR-133a-3p	7.3	1.4	2.51E-07	2.26E-05	hsa-miR-451a	5.5	1.1	2.33E-06	4.74E-05
hsa-miR-3648	7.1	1.3	1.50E-08	2.70E-06	hsa-miR-1246	5.4	1.1	7.44E-07	1.84E-05
hsa-miR-4792	6.4	1.2	9.79E-08	1.18E-05	hsa-miR-6892-5p	5.4	0.9	2.73E-08	1.05E-06
hsa-miR-134-5p	6.3	1.4	1.74E-05	5.69E-04	hsa-miR-6858-5p	5.0	1.5	1.11E-03	8.19E-03
hsa-miR-122-5p	5.9	1.3	4.20E-06	2.16E-04	hsa-miR-148a-3p	4.9	0.6	9.71E-15	8.67E-13
hsa-miR-663b	5.8	1.5	7.49E-05	1.80E-03	hsa-miR-760	4.8	1.0	7.81E-06	1.29E-04
hsa-miR-654-5p	5.8	1.5	1.23E-04	2.47E-03	hsa-miR-365a-5p	4.3	0.8	8.07E-08	2.79E-06
hsa-miR-4516	5.7	1.4	6.63E-05	1.80E-03	hsa-miR-4669	4.2	1.3	1.45E-03	9.67E-03
hsa-miR-381-3p	5.1	1.1	2.53E-06	1.52E-04	hsa-miR-378a-3p	4.2	1.0	3.96E-05	5.71E-04
hsa-miR-432-5p	5.1	1.6	5.71E-04	9.78E-03	hsa-miR-139-5p	4.1	1.3	1.33E-03	9.13E-03
hsa-miR-206	4.9	1.1	8.70E-06	3.48E-04	hsa-miR-541-5p	4.0	1.4	4.64E-03	2.35E-02
hsa-miR-145-3p	4.8	1.7	4.05E-03	4.70E-02	hsa-miR-150-5p	3.7	1.5	6.25E-03	3.00E-02
hsa-miR-143-3p	4.8	1.0	2.20E-06	1.52E-04	hsa-miR-1307-5p	3.5	0.7	4.21E-07	1.12E-05
hsa-miR-127-3p	4.7	1.2	7.40E-05	1.80E-03	hsa-miR-193b-5p	3.5	0.7	3.58E-06	6.89E-05
hsa-miR-382-5p	4.6	1.4	1.59E-03	2.48E-02	hsa-miR-378i	3.4	1.2	3.56E-03	1.98E-02
hsa-miR-199a-3p	4.4	1.0	8.10E-06	3.48E-04	hsa-miR-183-5p	3.3	0.9	3.13E-04	2.85E-03
hsa-miR-4508	4.3	1.4	2.80E-03	3.88E-02	hsa-miR-378a-5p	3.2	1.2	8.18E-03	3.63E-02
hsa-miR-199b-3p	4.3	1.0	1.12E-05	4.04E-04	hsa-miR-412-5p	3.2	0.8	7.73E-05	8.36E-04
hsa-miR-411-5p	4.0	1.3	2.47E-03	3.55E-02	hsa-miR-6807-5p	3.2	1.3	9.96E-03	4.15E-02
hsa-miR-100-5p	3.9	1.0	1.18E-04	2.47E-03	hsa-miR-486-3p	3.2	0.9	2.37E-04	2.28E-03
hsa-miR-199a-5p	3.5	0.9	1.85E-04	3.50E-03	hsa-miR-129-5p	3.1	0.9	2.95E-04	2.76E-03
hsa-miR-214-3p	3.5	1.2	3.91E-03	4.70E-02	hsa-miR-320d	3.1	1.1	6.42E-03	3.04E-02
hsa-miR-23a-3p	3.4	1.2	3.24E-03	4.17E-02	hsa-miR-365b-5p	3.0	0.7	5.95E-06	1.08E-04
hsa-let-7b-5p	3.2	0.9	4.05E-04	7.29E-03	hsa-miR-204-3p	2.9	1.1	9.48E-03	4.05E-02
hsa-miR-22-3p	2.5	0.8	3.13E-03	4.17E-02	hsa-miR-1304-3p	2.9	0.9	1.64E-03	1.03E-02
hsa-miR-103a-3p	-2.5	0.9	3.84E-03	4.70E-02	hsa-miR-431-3p	2.7	0.9	3.93E-03	2.12E-02
hsa-miR-128-3p	-2.9	0.9	1.90E-03	2.85E-02	hsa-miR-134-5p	2.7	0.7	1.86E-04	1.84E-03
hsa-miR-615-3p	-4.6	1.1	5.04E-05	1.51E-03	hsa-miR-486-5p	2.6	0.8	1.56E-03	1.00E-02
hsa-miR-4521	-4.6	1.2	1.03E-04	2.32E-03	hsa-miR-432-5p	2.6	0.6	7.28E-05	8.12E-04
hsa-miR-130b-5p	-4.7	1.4	1.24E-03	2.02E-02	hsa-miR-3158-3p	2.6	0.8	2.66E-03	1.56E-02
					hsa-miR-23a-5p	2.5	0.9	6.98E-03	3.14E-02
					hsa-miR-140-3p	2.4	0.6	7.19E-05	8.12E-04
					hsa-miR-320c	2.2	0.7	3.24E-03	1.84E-02
					hsa-miR-425-3p	2.2	0.7	1.75E-03	1.08E-02
					hsa-miR-19b-3p	2.2	0.8	4.22E-03	2.23E-02
					hsa-miR-106b-3p	2.1	0.7	2.20E-03	1.33E-02
					hsa-miR-501-3p	2.1	0.8	1.04E-02	4.28E-02
					hsa-miR-192-5p	2.0	0.6	1.53E-03	9.98E-03
					hsa-miR-500a-3p	1.9	0.8	1.07E-02	4.34E-02
					hsa-miR-125a-3p	1.9	0.7	4.25E-03	2.23E-02
					hsa-miR-625-3p	1.9	0.7	1.17E-02	4.70E-02
					hsa-miR-22-5p	1.8	0.7	6.70E-03	3.07E-02
					hsa-miR-24-3p	1.8	0.5	1.08E-04	1.14E-03
					hsa-miR-25-3p	1.8	0.4	6.57E-05	7.97E-04
					hsa-miR-185-5p	1.6	0.5	6.68E-04	5.50E-03
					hsa-miR-574-3p	1.6	0.6	5.96E-03	2.91E-02
					hsa-miR-193a-5p	1.5	0.6	6.63E-03	3.07E-02
					hsa-miR-218-5p	1.5	0.4	1.53E-04	1.55E-03
					hsa-miR-21-5p	-1.5	0.3	1.41E-05	2.12E-04
					hsa-miR-423-3p	-1.5	0.4	5.13E-04	4.33E-03
					hsa-miR-199a-5p	-1.6	0.4	6.68E-05	7.97E-04
					hsa-miR-99b-5p	-1.6	0.5	9.48E-04	7.29E-03
					hsa-miR-127-3p	-1.6	0.6	5.13E-03	2.54E-02
					hsa-miR-379-5p	-1.6	0.5	3.28E-04	2.91E-03
					hsa-miR-654-3p	-1.7	0.5	1.35E-03	9.13E-03
					hsa-miR-191-5p	-1.8	0.6	1.28E-03	9.13E-03
					hsa-miR-487b-3p	-1.9	0.8	1.24E-02	4.93E-02
					hsa-miR-494-3p	-2.0	0.6	8.61E-04	6.93E-03
					hsa-miR-31-5p	-2.1	0.5	4.48E-05	5.96E-04
					hsa-miR-221-5p	-2.1	0.5	4.19E-05	5.79E-04
					hsa-miR-155-5p	-2.2	0.7	3.20E-03	1.84E-02
					hsa-miR-28-5p	-2.2	0.8	4.68E-03	2.35E-02
					hsa-miR-23b-3p	-2.4	0.7	9.32E-04	7.29E-03
					hsa-miR-424-3p	-2.4	0.7	1.34E-03	9.13E-03
					hsa-miR-98-5p	-2.6	0.8	1.04E-03	7.82E-03
					hsa-miR-30c-2-3p	-2.6	0.9	2.24E-03	1.33E-02
					hsa-miR-484	-2.6	0.7	6.63E-05	7.97E-04
					hsa-miR-505-3p	-2.6	0.9	3.60E-03	1.98E-02
					hsa-miR-125a-5p	-2.6	0.6	7.11E-06	1.23E-04
					hsa-miR-374b-5p	-2.7	1.0	6.74E-03	3.07E-02
					hsa-miR-103a-3p	-2.9	0.5	4.33E-10	2.14E-08
					hsa-miR-7641	-3.0	1.2	9.10E-03	3.93E-02
					hsa-miR-125b-5p	-3.1	0.5	5.27E-11	3.65E-09
					hsa-miR-222-3p	-3.3	0.4	1.00E-14	8.67E-13
					hsa-miR-671-3p	-3.9	0.8	9.37E-07	2.16E-05
					hsa-miR-130b-5p	-4.1	0.9	1.15E-06	2.50E-05
					hsa-let-7a-5p	-4.1	0.6	1.54E-10	8.89E-09
					hsa-miR-455-3p	-4.2	1.0	8.41E-06	1.32E-04
					hsa-let-7e-5p	-4.3	0.8	1.81E-07	5.22E-06
					hsa-miR-411-5p	-4.3	0.5	5.98E-18	1.03E-15
					hsa-miR-335-3p	-4.7	0.9	1.19E-07	3.75E-06
					hsa-miR-411-3p	-5.3	0.9	3.23E-09	1.40E-07

Supplementary Table S3. Differentially expressed miRNAs in EV *vs* Non-EV samples.

EV *versus* non-EV (part 1)									
DE miRNA name	HEK				DE miRNA name	MSC			
	log2FC	lfcSE	pvalue	padj		log2FC	lfcSE	pvalue	padj
hsa-miR-7704	8.2	1.2	3.24E-11	1.43E-08	hsa-miR-1275	6.5	1.0	3.63E-11	2.80E-09
hsa-miR-6087	7.0	1.1	1.16E-09	2.56E-07	hsa-miR-128-1-5p	6.2	0.9	2.17E-11	2.34E-09
					hsa-miR-7977	5.9	0.9	1.40E-10	6.76E-09
					hsa-miR-503-3p	5.5	1.1	8.62E-07	1.75E-05
					hsa-miR-671-3p	5.4	0.8	4.42E-11	2.84E-09
					hsa-miR-370-5p	5.3	0.9	2.39E-08	7.08E-07
					hsa-miR-152-5p	5.3	0.9	8.19E-10	3.16E-08
					hsa-miR-5100	5.0	1.1	4.10E-06	6.89E-05
					hsa-miR-130b-5p	5.0	0.9	9.34E-09	3.00E-07
					hsa-miR-487b-5p	4.5	1.2	5.20E-05	4.67E-04
					hsa-miR-335-3p	4.4	0.9	1.13E-06	2.07E-05
					hsa-miR-323a-5p	4.4	1.0	1.25E-05	1.70E-04
					hsa-miR-148b-5p	4.4	1.1	3.11E-05	3.16E-04
					hsa-miR-23b-5p	4.2	1.0	4.37E-05	4.11E-04
					hsa-miR-329-5p	4.2	1.3	7.26E-04	4.06E-03
					hsa-miR-1225-3p	4.0	1.2	6.05E-04	3.60E-03
					hsa-miR-1343-3p	3.9	1.0	8.92E-05	7.49E-04
					hsa-miR-6789-3p	3.9	1.3	1.90E-03	8.81E-03
					hsa-miR-615-3p	3.9	0.9	2.49E-05	2.82E-04
					hsa-miR-92a-1-5p	3.8	0.9	2.20E-05	2.74E-04
					hsa-miR-6892-3p	3.7	1.4	6.00E-03	2.17E-02
					hsa-miR-410-5p	3.7	1.2	1.88E-03	8.81E-03
					hsa-miR-149-3p	3.6	1.2	2.34E-03	1.04E-02
					hsa-miR-221-5p	3.6	0.5	5.51E-12	1.06E-09
					hsa-miR-411-3p	3.6	1.0	1.17E-04	9.39E-04
					hsa-miR-29b-1-5p	3.6	1.1	1.17E-03	6.01E-03
					hsa-miR-92b-5p	3.5	0.6	3.22E-08	8.29E-07
					hsa-miR-30c-2-3p	3.5	0.9	5.56E-05	4.88E-04
					hsa-miR-30b-3p	3.5	1.2	3.98E-03	1.62E-02
					hsa-miR-4443	3.5	1.0	6.06E-04	3.60E-03
					hsa-miR-25-5p	3.5	0.8	2.70E-05	2.98E-04
					hsa-miR-671-5p	3.4	0.7	2.60E-07	5.57E-06
					hsa-let-7a-5p	3.4	0.6	1.23E-07	2.98E-06
					hsa-miR-6807-5p	3.3	1.0	1.74E-03	8.41E-03
					hsa-miR-6724-5p	3.3	1.2	4.57E-03	1.78E-02
					hsa-miR-433-5p	3.3	1.2	8.37E-03	2.80E-02
					hsa-miR-455-3p	3.2	1.0	1.04E-03	5.57E-03
					hsa-miR-27b-5p	3.1	1.1	4.92E-03	1.89E-02
					hsa-let-7e-5p	3.0	0.8	1.80E-04	1.29E-03
					hsa-miR-33b-3p	3.0	1.2	6.93E-03	2.39E-02
					hsa-miR-1908-5p	3.0	0.8	1.77E-04	1.29E-03
					hsa-miR-411-5p	2.9	0.5	6.10E-09	2.14E-07
					hsa-miR-485-3p	2.9	0.9	6.31E-04	3.63E-03
					hsa-miR-3187-3p	2.9	1.0	3.90E-03	1.60E-02
					hsa-let-7a-2-3p	2.9	0.9	2.03E-03	9.16E-03
					hsa-miR-365b-5p	2.8	0.6	1.28E-05	1.70E-04
					hsa-miR-23a-5p	2.7	0.8	1.32E-03	6.51E-03
					hsa-miR-484	2.7	0.7	5.92E-05	5.07E-04
					hsa-miR-222-3p	2.7	0.4	1.85E-10	7.92E-09
					hsa-miR-485-5p	2.7	0.7	1.07E-04	8.77E-04
					hsa-miR-409-5p	2.7	0.7	2.96E-04	1.97E-03
					hsa-miR-142-5p	2.6	1.0	1.06E-02	3.43E-02
					hsa-miR-193b-5p	2.6	0.7	1.49E-04	1.13E-03

Supplementary Table S3 (continued)

EV versus non-EV (part 2)									
DE miRNA name	HEK				DE miRNA name	MSC			
	log2FC	lfcSE	pvalue	padj		log2FC	lfcSE	pvalue	padj
					hsa-miR-1301-3p	2.6	1.1	1.57E-02	4.78E-02
					hsa-miR-365a-5p	2.5	0.7	6.43E-04	3.65E-03
					hsa-miR-31-5p	2.4	0.5	4.50E-06	7.24E-05
					hsa-miR-6892-5p	2.4	0.6	1.98E-04	1.39E-03
					hsa-miR-98-5p	2.4	0.8	2.80E-03	1.20E-02
					hsa-miR-654-5p	2.4	0.5	2.17E-06	3.81E-05
					hsa-miR-196b-5p	2.3	0.7	7.99E-04	4.41E-03
					hsa-miR-193a-5p	2.3	0.6	4.97E-05	4.56E-04
					hsa-miR-155-5p	2.3	0.8	3.10E-03	1.32E-02
					hsa-miR-1307-5p	2.2	0.6	5.30E-04	3.24E-03
					hsa-miR-92b-3p	2.1	0.6	9.81E-04	5.33E-03
					hsa-miR-1307-3p	2.1	0.5	1.37E-04	1.06E-03
					hsa-miR-92a-3p	2.1	0.5	1.61E-04	1.19E-03
					hsa-miR-103a-3p	2.1	0.5	9.01E-06	1.34E-04
					hsa-miR-191-5p	2.0	0.6	3.45E-04	2.22E-03
					hsa-miR-125b-5p	2.0	0.5	2.45E-05	2.82E-04
					hsa-miR-424-3p	1.9	0.8	1.24E-02	3.87E-02
					hsa-miR-23b-3p	1.9	0.7	8.01E-03	2.71E-02
					hsa-miR-125a-5p	1.9	0.6	1.30E-03	6.51E-03
					hsa-miR-24-2-5p	1.8	0.7	1.55E-02	4.74E-02
					hsa-miR-328-3p	1.8	0.7	1.38E-02	4.25E-02
					hsa-miR-423-3p	1.6	0.4	2.29E-04	1.55E-03
					hsa-miR-196a-5p	1.6	0.6	6.13E-03	2.17E-02
					hsa-miR-433-3p	1.6	0.6	1.14E-02	3.60E-02
					hsa-miR-181b-5p	-1.6	0.5	4.95E-04	3.13E-03
					hsa-miR-99a-5p	-1.6	0.4	2.90E-05	3.02E-04
					hsa-miR-532-5p	-1.7	0.6	2.56E-03	1.12E-02
					hsa-miR-30e-5p	-1.8	0.7	1.11E-02	3.59E-02
					hsa-miR-767-5p	-1.8	0.6	5.32E-03	2.01E-02
					hsa-miR-339-3p	-1.8	0.6	3.33E-03	1.38E-02
					hsa-miR-218-5p	-1.8	0.4	8.68E-06	1.34E-04
					hsa-miR-30d-5p	-1.9	0.7	6.62E-03	2.30E-02
					hsa-miR-143-3p	-1.9	0.5	3.10E-04	2.03E-03
					hsa-miR-22-3p	-2.0	0.4	1.95E-07	4.43E-06
					hsa-miR-134-5p	-2.0	0.7	4.57E-03	1.78E-02
					hsa-miR-24-3p	-2.1	0.5	1.02E-05	1.46E-04
					hsa-miR-425-3p	-2.1	0.8	8.49E-03	2.80E-02
					hsa-miR-101-3p	-2.1	0.9	1.22E-02	3.84E-02
					hsa-miR-432-5p	-2.1	0.7	1.25E-03	6.35E-03
					hsa-miR-186-5p	-2.2	0.7	1.12E-03	5.90E-03
					hsa-miR-181a-5p	-2.2	0.5	1.83E-05	2.36E-04
					hsa-miR-320c	-2.3	0.8	5.44E-03	2.04E-02
					hsa-miR-105-5p	-2.4	0.9	6.55E-03	2.30E-02
					hsa-miR-382-3p	-2.4	0.8	1.80E-03	8.58E-03
					hsa-miR-22-5p	-2.5	0.8	1.14E-03	5.94E-03
					hsa-miR-148a-3p	-2.6	0.6	2.85E-05	3.02E-04
					hsa-miR-136-5p	-2.6	0.9	4.49E-03	1.78E-02
					hsa-miR-148b-3p	-2.7	0.6	2.44E-05	2.82E-04
					hsa-miR-192-5p	-2.7	0.7	1.29E-04	1.01E-03
					hsa-miR-29a-3p	-2.7	0.4	8.90E-11	4.91E-09
					hsa-miR-27a-3p	-2.8	0.4	2.43E-11	2.34E-09
					hsa-miR-410-3p	-2.8	1.0	7.66E-03	2.62E-02
					hsa-miR-345-5p	-2.9	1.0	5.92E-03	2.17E-02
					hsa-miR-376c-3p	-2.9	1.1	1.12E-02	3.59E-02
					hsa-miR-30a-5p	-3.0	0.6	1.04E-06	2.00E-05
					hsa-miR-21-3p	-3.2	0.8	4.17E-05	4.02E-04
					hsa-miR-381-3p	-3.2	0.6	2.68E-08	7.39E-07
					hsa-miR-193b-3p	-3.3	1.1	3.16E-03	1.33E-02
					hsa-miR-1246	-3.4	1.1	2.04E-03	9.16E-03
					hsa-miR-660-5p	-3.5	1.1	4.23E-03	1.70E-02
					hsa-miR-136-3p	-3.5	0.8	3.99E-05	3.94E-04
					hsa-miR-376a-3p	-3.5	1.2	5.76E-03	2.14E-02
					hsa-miR-424-5p	-3.7	1.4	8.45E-03	2.80E-02
					hsa-miR-377-3p	-4.0	1.2	1.47E-03	7.18E-03
					hsa-miR-548o-3p	-4.1	1.3	1.92E-03	8.81E-03
					hsa-miR-19b-3p	-4.1	1.0	2.15E-04	1.48E-03
					hsa-miR-760	-4.6	1.3	6.16E-04	3.60E-03
					hsa-miR-122-5p	-6.2	0.8	6.80E-15	2.62E-12
